# A New Mechanism of Supraventricular Tachycardia: Gene Mutation

**DOI:** 10.2174/011573403X320610250108113731

**Published:** 2025-01-20

**Authors:** Jie Gao, Rong Luo, Xiaoping Li

**Affiliations:** 1 Department of Geriatric Cardiovascular Disease, Sichuan Academy of Medical Sciences and Sichuan Provincial People’s Hospital, Chengdu, Sichuan, China;; 2 Institute of Geriatric Cardiovascular Disease, Chengdu Medical College, Chengdu, Sichuan, China;; 3 Department of Cardiology, Hospital of the University of Electronic Science and Technology of China, Sichuan Provincial People’s Hospital, Chengdu, Sichuan, China;; 4 Sichuan Provincial Key Laboratory for Human Disease Gene Study, Sichuan Provincial People's Hospital, University of Electronic Science and Technology of China, Chengdu, China

**Keywords:** Supraventricular tachycardia, genetic mutations, epidemiology, ion channel, signaling pathways, atrioventricular node reentrant tachycardia

## Abstract

**Background:**

Supraventricular tachycardia (SVT) is very common in daily clinical practice, especially in the emergency department, with rapid onset and urgent management. The review highlights the recent genetic predispositions and mechanisms in SVT.

**Methods:**

Through analysis of epidemiology, familial clustering, and gene mutations of the relevant literature, the review elucidates the genetic properties and potential pathophysiology of SVT.

**Results:**

There are many pathophysiological mechanisms related to atrioventricular node reentrant tachycardia (AVNRT) and atrioventricular reentrant tachycardia (AVRT). Currently, there is relatively little research on inappropriate sinus tachycardia (IST), atrial tachycardia (AT), and congenital junctional ectopic tachycardia (CJET). It seems that every type of SVT has gene mutations in ion channels, with three types of SVT having gene mutations in signaling pathways, and others including gene mutations in beta-adrenergic-receptor autoantibodies, autonomic nervous system, and AV node structure.

**Conclusion:**

SVT has certain genetic characteristics and is often associated with other heart diseases. From the analysis of mutated genes in SVT, it appears to be a type of cardiac ion channel disease. Unlike common ion channel diseases, it is more insidious and more susceptible to external factors. The confirmation of the genetic basis of SVT provides direction for future hazard stratification assessment and gene targeted therapy drug research.

## 1 INTRODUCTION

Supraventricular tachycardia (SVT) is an abnormally rapid heart rhythm originating above the ventricles, mostly with a narrow QRS complex. It conventionally includes atrioventricular node reentrant tachycardia (AVNRT), atrioventricular reentrant tachycardia (AVRT), sinus tachycardia, Atrial tachycardia (AT), and inappropriate sinus tachycardia (IST, without obvious heart disease or other cause for sinus tachycardia) [1]. And paroxysmal supraventricular tachycardia (PSVT) specifically refers to AVNRT and AVRT [including the Wolff-Parkinson-White (WPW) syndrome], which incidence is more than 90% SVT [2]. In addition, there is a rare type of SVT called congenital junctional ectopic tachycardia (CJET).

In recent years, the discovery of familial SVT suggests that genetic factors are involved in the occurrence and development of the disease. Several genetic characteristics have been associated with SVT. One of the most well-known gene mutations related to SVT is PRKAG2, which encodes a subunit of AMP-activated protein kinase (AMPK) [3, 4]. Besides PRKAG2, mutations in genes encoding cardiac ion channels, such as FLRT3 in the TGF-β/SMAD4 signaling pathway, have also been identified in individuals with SVT [5]. These mutations can disrupt the normal electrical signaling in the heart, leading to episodes of rapid heart rates characteristic of SVT.

## 2 EPIDEMIOLOGY

Survey data show that the prevalence of PSVT was 2.29% and the incidence was 36/100,000 person-years. Compared with patients with other cardiovascular diseases, patients with PSVT had a younger age (mean 37 *versus* 69 years; *P*= 0. 0002) and females seem to be more likely to suffer from PSVT than males (2 times more than males). Most of IST patients are young and female [6]. A single-center cohort study found that 92 percent of IST patients were female, with an average age of 33 years [7]. Focal AT is relatively uncommon, accounting for 5% to 15% in adults undergoing study for SVT. There seemed to be no difference in prevalence between males and females [8]. CJET patients may manifest within the first few days to weeks after birth or even appear at birth [9]. Without timely diagnosis and proper treatment, it will lead to a high incidence rate and mortality of up to 35% [10].

## 3 AVNRT

### 3.1 AVNRT Familial Clustering

In 2004, Hayes *et al*. first reported 6 cases of familial AVNRT, in which 3 families had AVNRT with both their probands and their daughters, and proposed autosomal dominant (AD) as its most likely inheritance pattern, laying the genetic basis for AVNRT [11]. In 2006, Frisch *et al*. reported two siblings with Wolfram syndrome (WFS) accompanied by AVNRT, which encoded transmembrane glycoproteins caused by mutations in the Wolframin protein gene [12]. The protein had important physiological functions in membrane trafficking, secretion, processing, and regulation of endoplasmic reticulum calcium homeostasis [13-15], and it was speculated that WFS1 gene might be a pathogenic gene for AVNRT [16]. In 2012, Namgung *et al*. proposed the mother-son inheritance approach of familial AVNRT lineage, suggesting that the pattern of inheritance may be autosomal dominant, autosomal recessive (AR), or X-linked, but genetic testing was not performed in this study to further clarify the inheritance pattern [17]. In 2013, two 17-year-old identical twins were reported to suffer from AVNRT at the same time, which also confirmed the heritability of AVNRT [18]. In 2015, Stec *et al*. discovered the world's largest hereditary AVNRT-six female patients in the same family and speculated that the inheritance mode may be AD, X-linked dominant, or mitochondrial carry [19]. However, in 2017, familial AVNRT in the father-son inheritance pattern suggested that the X-linked inheritance pattern was less likely [20]. In 2017, Michowitz *et al*. found that the prevalence of familial AVNRT was not very little in patients undergoing radiofrequency catheter ablation, with a prevalence of 127/10000 (95% CI 82~196/10000), and for patients with AVNRT, the prevalence in first-degree relatives was 3.6 times higher than in the general population [21]. In 2021, our team reported familial hereditary AVNRT in 8 families and speculated that the inheritance mode was perhaps AD or X-linked [22]. Overall, the incidence rate of familial AVNRT is as high as 127/10000 (95% CI 82-196/10000), and the incidence rate of first-degree relatives is 3.6 times higher than that of the general population, with early onset age and no organic heart disease [21].

### 3.2 Pathophysiology of Familial AVNRT

Andreasen *et al*. selected 298 AVNRT patients with 67 sequenced genes, and found that 26.4% of patients had one or more gene mutations that affected the function of sodium channels (such as SCN5A, SCN1B, SCN5A, and SCN1B), 19% of patients had genetic mutations that affected calcium ionic influx (RYR2, RYR3, CACNB2, and CACNAID), and 4.3% of patients had gene mutations in hyperpolarization-activated cyclic nucleotide gating channels (HCN-4) [23]. Recently, 82 patients with sporadic AVNRT were detected by whole-exome sequencing (WES), revealing 126 rare mutations (allele frequency < 0.001) in 48 genes, and 10 candidate pathogenic genes were screened for the first time, which were RYR2, NOS1, SCN1A, CFTR, EPHB4, ROBO1, PRKAG2, MMP2, ASPH, and ABCC8, suggesting that pathways such as autonomic nervous system, neurotransmitter release, and cardiac contraction may be involved in AVNRT, as well as ion channels [22]. There is a study speculating that mutations in cardiomyocytegenes(TTN, NKX2-5, and MYH6)are involved in AVNRT [24], and only NKX2-5 has been confirmed to be related to bundles of conductive tissue between the atria and ventricles [25]. Studies have revealed that mutations in SCN5A, SCN10A, and SCN1B in patients with AVNRT can lead to the inactivation of voltage-gated sodium channels, thereby reducing the excitability and conduction velocity of myocardial cells, prolonging the refractory period of some myocardial cells, and providing a basis for the AVNRT turnaround pathway [26]. Meanwhile, in patients with this type of tachycardia, other abnormalities such as Brugada syndrome, atrial fibrillation, and left anterior branch block are often present simultaneously [27, 28]. Besides SCN5A, SCN10A, and SCN1B, mutations related to sodium ion channels such as GPD1L, PKP2, and HEY2 may also lead to a decrease in the functional excitability of sodium ions, resulting in blockage of the atrioventricular (AV) node pathway and the occurrence of reentrant excitability [26]. Podliesna *et al*. studied the genes of three independent PSVT family members accompanied by conduction block, dilated cardiomyopathy, and sudden death-related diseases or not, with the same gene mutations in the cardiac troponin I-interacting kinase (TNNI3K) gene (c.2302G>A, p. Glu768Lys) which led to increased conduction indices by increased troponin I interaction kinase activity in a series of signal path changes [29, 30]. Taken together, these studies provide further evidence that AVNRT may be associated with abnormal mutations in sodium, calcium channels, other ion channel genes, structural abnormalities and dysregulation of the autonomic nervous system, as well as abnormal signaling pathways due to mutations in enzymes.

## 4 AVRT

### 4.1 AVRT Familial Clustering

The true prevalence of concealed accessory pathways is not clear, since only patients with symptomatic arrhythmias can diagnose concealed accessory pathways, the prevalence among patients with SVT presenting for catheter ablation is approximately 15% [31-33]. In 1998, a sister and brother were cured by catheter ablation (CA), who both suffered from concealed accessory pathways, supporting a potential genetic problem during the development of fetal AV connections [34]. Among patients with WPW syndrome, preexcitation syndrome was found to be present in 3.4% of first-degree relatives [35]. In 1944, Ohnell first reported 2 cases of familial WPW syndrome and confirmed the existence of AVRT [36]. With more and more research on the genetic aspects of preexcitation syndrome, familial WPW syndrome was usually considered to be inherited as an AD trait [3, 37, 38].

### 4.2 Pathophysiology of Familial AVRT

**Fig. (1):**
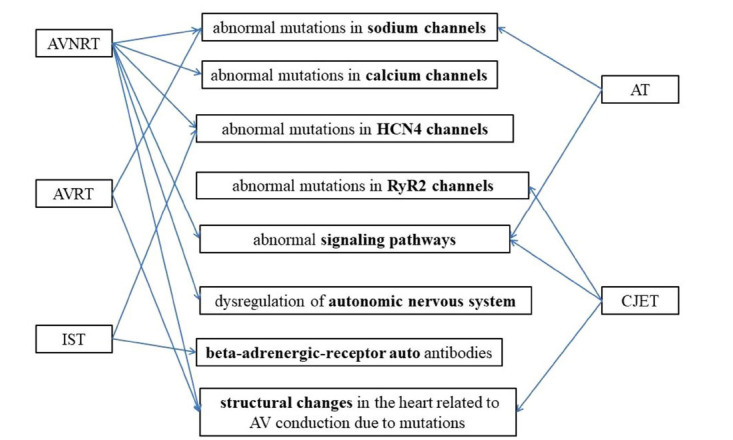
Pathophysiology of familial supraventricular tachycardia.

Genetic animal models of WPW that express the PRKAG2 gene mutation encoding AMPK have been developed to be consistent with the phenotype of human WPW syndrome [39]. Missense mutations were identified in the genes encoding the PRKAG2 and LAMP2, which had been associated with left ventricular hypertrophy(LVH)in association with WPW syndrome [3, 40-43]. In a study of 24 patients with LVH and WPW, 29% had PRKAG2 mutations, and 16% had LAMP2 mutations [44]. But the PRKAG2 mutation in isolated LVH syndrome is only 0.5% [41]. Moreover, the R302Q mutation in PRKAG2 has been proven to be associated with Mahaim-associated preexcitation syndrome [45]. The PRKAG2 gene encodes the AMPK γ 2 regulatory subunit [3], which is a metabolic sensor for ATP storage and can provide ATP according to the energy requirements of cells [46]. It inhibits glycogen synthase, thereby increasing the energy production availability of glucose [47]. Cardiac glycogen deposition was observed in a transgenic model expressing the N488I missense mutation in the PRKAG2 gene [48]. Light *et al*. documented the slowing down of sodium channel open-state inactivation and prolonged action potential duration caused by AMPK R302Q mutation, indicating that cardiac ion channels may be substrates of AMPK [49]. The heart is unable to form an AV insulating state during embryonic development due to AMPK dysfunction which results in residual part of the AV junction,or glycogen accumulation promotes cell coupling and accelerates conduction. Mutations in the LAMP2 gene may have a similar pathogenesis to PRKAG2 heart syndrome, as both cause glycogen-accumulating cardiomyopathy [44]. Microdeletion of the BMP2 gene encoding bone morphogenetic protein-2 is identified to affect the development of annulus fibrosus in WPW patients [50]. It seems that gene mutations associated with AVRT mostly caused structural changes in the heart related to AV conduction. The underlying pathophysiological mechanisms that cause certain phenotypes of WPW are still unclear, but maybe we can try to analyze it in a way that combines the whole exome sequencing in conjunction with cell electrophysiological function in the future [51] (Fig. **1**).

## 5 IST

### 5.1 IST Familial Clustering

Genetic characteristics of IST can explain the occurrence of this condition within members of the same family. In 1941, Wising first described a familial form of IST that was labeled as familial, congenital sinus tachycardia [52]. In 2015, four out of 48 IST patients were found to have a familial history [53]. In 2016, a familial form of IST was reported to be associated with a R524Q gain-of-function mutation in the HCN4 channels [54].

### 5.2 Pathophysiology of Familial IST

The etiology of familial IST may related to abnormal ion channels in the sinus node and beta-adrenergic-receptor autoantibodies [55-57]. HCN channels have been confirmed to be related to cardiac pacemaker cells [58]. Among them, HCN4 is the most common HCN channel in the human sinoatrial node [6]. A functional gain mutation (arginine glutamine; R524Q) occurred in the HCN4 gene in family IST patients, which imitates beta-adrenergic stimulation to increase the funny current by increasing sensitivity to cyclic adenosine monophosphate (cAMP), leading to an increased firing rate of pacemaker cells [53, 54]. Chiale *et al*. used COS-7 cells transfected with genes encoding for β1 or β2 adrenergic receptors and found that antibodies in the circulation β-Adrenergic IgG antibodies, which produce positive heart rate promoting effects by increasing cAMP levels [57, 59]. Excessive sympathetic or decreased parasympathetic excitability, excessive intrinsic HR, dysfunctional neurohormonal modulation, and ectopicactivity of the sinoatrial node are also associated with the occurrence of IST but have not been reported in familial IST [60-62]. Although genetic mutations causing abnormal cardiac electrical activity are important in family IST, other external triggering factors such as stress, caffeine, medications, or other medical conditions may also greatly affect the onset and severity of IST [63].

## 6 AT

### 6.1 AT Familial Clustering

In 2023, a 14-year-old AT patient with prolonged HV interval first reported mutations in genes encoding cardiac ion channels [64]. In 2019, Podliesna *et al*. isolated the same gene genetic variant in three independent multigenerational families, all of which manifested as AT with or without DCM and sudden death [29]. In 2019, a newborn female infant with congenital myopathy was reported to have persistent AT associated with RYR1 gene mutations [65]. In 2014, Theis *et al*. used linkage analysis and an exome sequencing approach and found a family with AT, dilated cardiomyopathy and conduction system disease [66]. Dagres *et al*. characterized autosomal dominant familial AT without structural heart disease in 2004 [67]. The familial AT reported by Balaji *et al*. in 1996 presented in male family members who showed symptoms during the neonatal period [68]. Brodsky *et al*. described a familial AT with a short PR interval, and most of the patients co-ordinated with paroxysmal or chronic atrial fibrillation [69].

### 6.2 Pathophysiology of Familial AT

Similar to AVNRT, mutations in TNNI3K are also found in family members of patients with AT [70] . The mutation of TNNI3K, which expresses functional amino acid kinases with cardiac restricted expression patterns, does not directly affect the functional sites in the catalytic domain of ATP binding, but it has an effect on the assembly of proteins through certain signaling pathways such as MAPKs signaling pathway, mitochondrial signaling pathway, which ultimately leads to changes in a series of sarcometylum contraction regulatory proteins such as cardiac troponin I and cardiac actin, and ultimately promotes the occurrence of AT [71]. Mutations in the SCN5A p.R367H variant with overlapping presentations can lead to dysfunction of cardiac sodium voltage-gated channel, which may partly explain the occurrence of AT [64, 72]. The possible mechanism is that the tetrodotoxin-resistant sodium channel encoded by SCN5A may inhibit the normal atrial conduction pathway [73-75]. Analysis reveals that AT with genetic characteristics is often accompanied by prolonged HV interval, sick sinus syndrome, and other abnormalities in the cardiac conduction system.

## 7 CJET

### 7.1 CJET Familial Clustering

CJET is another rare type of SVT that occurs in newborns and infants originating from the AV junction of the heart,. CJET is considered a congenital illness, meaning it is present at birth with a high fatality rate as high as 35% [76]. Nearly half of CJET patients can detect a positive family history [77, 78]. Dubin *et al*. described three families associated with anti-SSA and anti-SSB antibodies detected in both mothers and their CJET children. In 2015, the TNNI3K gene was identified in a family as a cause of JET accompanied by familial conduction system disease [79].

### 7.2 Pathophysiology of CJET

Genes related to signaling pathways, ion channels, and structural abnormalities in the cardiac conduction system may contribute to the pathophysiology of CJET. TNNI3K is currently the only gene known to be specifically expressed in the heart, encoding the MAP kinase that interacts with the cardiac troponin I in the signaling pathways [80]. The mutation of goalkeeper Thr in TNNI3K may ultimately lead to abnormal phosphorylation of its substrate cardiac troponin I and might result in CJET [79, 81-83]. Jph2-encoded junctophilin-2 is a structural protein that maintains normal excitation contraction coupling and regulates Ca^2+^ release by the sarcoplasmic reticulum (SR) through direct interaction with ryanodine type 2 receptor (RyR2) in cardiac muscle cells [84-86]. The downregulation of Jph2 has been shown to increase Ca^2+^ leakage from the local myocardial cell SR pool, leading to CJET [87, 88]. Inherited studies on CJET supposed partial injury to AV node conduction system, in which automaticity was enhanced [78]. This abnormal automaticity leads to rapid and disorganized depolarization of the cardiac tissue in the vicinity of the AV junction. At the same time, the AV node injury in CJET has been confirmed to potentially lead to a secondary conduction block of the heart by autopsy examination [76].

## 8 DISCUSSION

At present, the research on SVT gene is constantly deepening, and many studies mainly focus on familial AVNRT and WPW syndrome, while the genetics of occult bypass, IST, AT and CJET are relatively few. SVT seems to be a disease characterized by abnormal cardiac ion channels and cascade reactions of cardiac protein kinase signaling. In the heart, voltage-gated ion channels are responsible for the generation and propagation of action potentials in most excitable tissues [89]. Ionic channels are crucial for the orderly progression of action potentials, and mutations in genes related to cardiac tissue electrical excitability can trigger SVT [90, 91]. Protein kinases in myocardial cells are usually activated under the stimulation of biomechanical tension or remodeling factors [92]. Activated protein kinases can directly phosphorylate sarcomere structural proteins such as cardiac troponin I (cTnI), cardiac troponin T (cTnT), myosin binding protein C (MyBPC), etc., affecting myocardial cell contractility and leading to arrhythmia [93, 94]. Studies focusing on SVT-related key mutated genes and mechanisms are summarized in Table **1**.

## CONCLUSION

At present, the main treatment for AVT is radiofrequency ablation, which can only be detected after the onset of the patient. However, there are potential risks if the patient is engaged in high-risk work, such as driving and working at heights when the attack occurs. Although currently understanding the pathogenic genes of SVT patients does not alter the treatment process, genetic testing can provide prognostic data for the proband and their relatives. It can predict the possible onset of diseases and related clinical outcomes for the relatives of the proband while also facilitating clinical doctors to pay more attention to this group of patients. These advances will provide risk assessment and early electrophysiological examination for high-risk populations. Moreover, the confirmation of the SVT gene mutations may provide new antiarrhythmic molecular targeted drugs.

## Figures and Tables

**Table 1:** SVT-related key mutated genes and mechanisms.

**SVT**	**Gene**	**Pathways**	**Mechanism**	**References**
AVNRT	SCN5A, SCN10A, SCN1B	Sodium channels	Reducing the excitability and conduction velocity of myocardial cells, prolonging the refractory period of some myocardial cells.	[26-28]
	GPD1L, PKP2, HEY2	Sodium channels	Blockage of the AV node pathway and the occurrence of reentrant excitability.	[26]
	TNNI3K	Signaling pathways	Increased conduction indices by increased troponin I interaction kinase activity.	[29]
AVRT	PRKAG2	Sodium channels	The slowing down of sodium channel open state inactivation and prolonged action potential duration caused by AMPK mutation, unable to form an AV insulating state during embryonic development.	[43, 48]
IST	HCN4	HCN channels	Increasing sensitivity to cAMP, leading to an increased firing rate of pacemaker cells.	[52, 53]
AT	TNNI3K	Signaling pathways	Leading to changes in a series of sarcometylum contraction regulatory proteins, such as cardiac troponin I and cardiac actin.	[71]
	SCN5A	Sodium channels	The tetrodotoxin-resistant sodium channel encoded by SCN5A inhibiting the normal atrial conduction pathway.	[64, 72]
CJET	TNNI3K	Signaling pathways	The mutation of goalkeeper Thr in TNNI3K leads to abnormal phosphorylation of its substrate cardiac troponin I.	[79, 81]
	Jph2	RyR2 channels	The downregulation of Jph2 increases Ca^2+^ leakage from the local myocardial cell SR pool.	[87, 88]

**Abbreviations:** SVT, supraventricular tachycardia; AVNRT, atrioventricular node reentrant tachycardia; AVRT, atrioventricular reentrant tachycardia; IST, inappropriate sinus tachycardia; AT, Atrial tachycardia; CJET, congenital junctional ectopic tachycardia; AMPK, AMP-activated protein kinase; AV, atrioventricular; cAMP, cyclic adenosine monophosphate; HCN, hyperpolarization-activated cyclic nucleotide gating channels; RyR2, ryanodine type 2 receptor; SR, sarcoplasmic reticulum.

## LIST OF ABBREVIATIONS

AT: Atrial Tachycardia
AV: Atrioventricular
AVNRT: Atrioventricular Node Reentrant Tachycardia
AVRT: Atrioventricular Reentrant Tachycardia
CJET: Congenital Junctional Ectopic Tachycardia
IST: Inappropriate Sinus Tachycardia
RyR2: Ryanodine Type 2 Receptor

## References

[1] Josephson M.E., Wellens H.J.J. (1990). Differential diagnosis of supraventricular tachycardia.. Cardiol. Clin..

[2] Zaiti A.S.S., Magdic K.S. (2016). Paroxysmal supraventricular tachycardia.. Crit. Care Nurs. Clin. North Am..

[3] Gollob M.H., Green M.S., Tang A.S.L., Gollob T., Karibe A., Hassan A-S., Ahmad F., Lozado R., Shah G., Fananapazir L., Bachinski L.L., Tapscott T., Gonzales O., Begley D., Mohiddin S., Roberts R. (2001). Identification of a gene responsible for familial Wolff-Parkinson-White syndrome.. N. Engl. J. Med..

[4] Wolf C.M., Arad M., Ahmad F., Sanbe A., Bernstein S.A., Toka O., Konno T., Morley G., Robbins J., Seidman J.G., Seidman C.E., Berul C.I. (2008). Reversibility of PRKAG2 glycogen-storage cardiomyopathy and electrophysiological manifestations.. Circulation.

[5] Pang Y., Xu Y., Chen Q., Cheng K., Ling Y., Jang J., Ge J., Zhu W. (2024). FLRT3 and TGF ‐β/ SMAD4 signalling: Impacts on apoptosis, autophagy and ion channels in supraventricular tachycardia.. J. Cell. Mol. Med..

[6] Ali M., Haji A.Q., Kichloo A., Grubb B.P., Kanjwal K. (2021). Inappropriate sinus tachycardia: A review.. Rev. Cardiovasc. Med..

[7] Shabtaie S.A., Witt C.M., Asirvatham S.J. (2020). Natural history and clinical outcomes of inappropriate sinus tachycardia.. J. Cardiovasc. Electrophysiol..

[8] Chen S.A., Chiang C.E., Yang C.J., Cheng C.C., Wu T.J., Wang S.P., Chiang B.N., Chang M.S. (1994). Sustained atrial tachycardia in adult patients. electrophysiological characteristics, pharmacological response, possible mechanisms, and effects of radiofrequency ablation.. Circulation.

[9] Memon D., Larkin E., Varghese M. (2022). Congenital junctional ectopic tachycardia in the paediatric emergency department.. Cardiol. Young.

[10] Ashraf, M, Goyal A. (April 7, 2023). Junctional Ectopic Tachycardia.. In: StatPearls.

[11] Hayes J.J., Sharma P.P., Smith P.N., Vidaillet H.J. (2004). Familial atrioventricular nodal reentry tachycardia.. Pacing Clin. Electrophysiol..

[12] Strom T., Hörtnagel K., Hofmann S., Gekeler F., Scharfe C., Rabl W., Gerbitz K.D., Meitinger T. (1998). Diabetes insipidus, diabetes mellitus, optic atrophy and deafness (DIDMOAD) caused by mutations in a novel gene (wolframin) coding for a predicted transmembrane protein.. Hum. Mol. Genet..

[13] Smith C.J.A., Crock P.A., King B.R., Meldrum C.J., Scott R.J. (2004). Phenotype-genotype correlations in a series of wolfram syndrome families.. Diabetes Care.

[14] Hofmann S., Philbrook C., Gerbitz K.D., Bauer M.F. (2003). Wolfram syndrome: Structural and functional analyses of mutant and wild-type wolframin, the WFS1 gene product.. Hum. Mol. Genet..

[15] Osman A.A., Saito M., Makepeace C., Permutt M.A., Schlesinger P., Mueckler M. (2003). Wolframin expression induces novel ion channel activity in endoplasmic reticulum membranes and increases intracellular calcium.. J. Biol. Chem..

[16] Frisch D.R., Kwaku K.F., Allocco D.J., Zimetbaum P.J. (2006). Atrioventricular nodal reentrant tachycardia in two siblings with Wolfram syndrome.. J. Cardiovasc. Electrophysiol..

[17] Namgung J., Kwak J.J., Choe H., Kwon S.U., Doh J.H., Lee S.Y., Lee W.R. (2012). Familial occurrence of atrioventricular nodal reentrant tachycardia in a mother and her son.. Korean Circ. J..

[18] Barake W., Caldwell J., Baranchuk A. (2013). Atrioventricular nodal re-entry tachycardia in identical twins: A case report and literature review.. Indian Pacing Electrophysiol. J..

[19] Stec S., Deutsch K., Krajka Z.A. (2015). The world’s largest family with familial atrio-ventricular nodal reentry tachycardia.. Kardiol. Pol..

[20] Michowitz Y., Belhassen B. (2017). Response by michowitz and belhassen to letter regarding article, “familial occurrence of atrioventricular nodal reentrant tachycardia”.. Circ. Arrhythm. Electrophysiol..

[21] Michowitz Y., Heusler A.A., Reinstein E., Brodie T.O., Glick A., Belhassen B. (2017). Familial occurrence of atrioventricular nodal reentrant tachycardia.. Circ. Arrhythm. Electrophysiol..

[22] Chen X., Yan C., Luo R., Zhu Y., Qian M., Liu X., Liu M., Ikeda T., Li X. (2021). Clinical report of 8 families with atrioventricular nodal reentrant tachycardia from China.. Kardiol. Pol..

[23] Andreasen L., Ahlberg G., Tang C., Andreasen C., Hartmann J.P., Hansen T.J., Behr E.R., Pehrson S., Haunsø S., LuCamp, Weeke P.E., Jespersen T., Olesen M.S., Svendsen J.H. (2018). Next-generation sequencing of AV nodal reentrant tachycardia patients identifies broad spectrum of variants in ion channel genes.. Eur. J. Hum. Genet..

[24] Andreasen L., Ahlberg G., Ægisdottir H.M., Sveinbjörnsson G., Lundegaard P.R., Hartmann J.P., Müller P.C., Turdeghal H.K., Ghouse J., Pehrson S., Jensen H.K., Riahi S., Hansen J., Sandgaard N., Sørensen E., Banasik K., Sækmose S.G., Bruun M.T., Hjalgrim H., Erikstrup C., Pedersen O.B., Wittig M., Haunsø S., Ostrowski S.R., Franke A., Brunak S., Kanters J.K., Ellervik C., Bundgaard H., Ullum H., Gudbjartsson D.F., Thorsteinsdottir U., Holm H., Arnar D.O., Stefansson K., Svendsen J.H., Olesen M.S. (2022). Genetic variants close to *ttn*, *nkx2-5*, and *myh6* associate with avnrt.. Circ. Res..

[25] Aegisdottir H.M., Andreasen L., Thorolfsdottir R.B., Sveinbjornsson G., Jonsdottir A.B., Stefansdottir L., Thorleifsson G., Sigurdsson A., Halldorsson G.H., Barc J., Simonet F., Tragante V., Oddsson A., Ferkingstad E., Svendsen J.H., Ghouse J., Ahlberg G., Müller P.C., Turdeghal H.K., Bustamante M., Ulfarsson M.O., Helgadottir A., Gretarsdottir S., Saevarsdottir S., Jonsdottir I., Erikstrup C., Ullum H., Sørensen E., Brunak S., Jøns C., Zheng C., Bezzina C.R., Knowlton K.U., Nadauld L.D., Sulem P., Ostrowski S.R., Pedersen O.B., Arnar D.O., Gudbjartsson D.F., Olesen M.S., Bundgaard H., Holm H., Stefansson K., Banasik K., Bay J., Boldsen J.K., Brodersen T., Brunak S., Demur B.A., Christoffersen L.A.N., Didriksen M., Dinh K.M., Dowsett J., Erikstrup C., Feenstra B., Geller F., Gudbjartsson D., Hansen T.F., Mikkelsen H.D., Hindhede L., Hjalgrim H., Stemann J.H.V., Jensen B.A., Schork J.A., Kaspersen K., Kjerulff B.D., Kongstad M., Mikkelsen S., Mikkelsen C., Nissen I., Nyegaard M., Ostrowski S.R., Pedersen O.B., Quinn L.J.E., Rafnar Þ., Rohde P.D., Rostgaard K., Schwinn M., Stefansson K., Stefánsson H., Sørensen E., Thorsteinsdóttir U., Thørner L.W., Bruun T.M., Ullum H., Werge T., Westergaard D. (2024). Genome-wide association study of accessory atrioventricular pathways.. JAMA Cardiol..

[26] Hasdemir C., Payzin S., Kocabas U., Sahin H., Yildirim N., Alp A., Aydin M., Pfeiffer R., Burashnikov E., Wu Y., Antzelevitch C. (2015). High prevalence of concealed Brugada syndrome in patients with atrioventricular nodal reentrant tachycardia.. Heart Rhythm.

[27] Lee T.Y., Hogarth K., Szabo E., Maynes J.T. (2022). Sex‐specific arrhythmias caused by cardiac sodium channel na _v_ 1.5 mutation alters cardiomyocyte metabolism.. FASEB J..

[28] Vanninen S.U.M., Nikus K., Setälä A.K. (2017). Electrocardiogram changes and atrial arrhythmias in individuals carrying sodium channel *SCN5A D1275N* mutation.. Ann. Med..

[29] Podliesna S., Delanne J., Miller L., Tester D.J., Uzunyan M., Yano S., Klerk M., Cannon B.C., Khongphatthanayothin A., Laurent G., Bertaux G., Eicher F.S., Wu S., Yen H.Y., Gao H., Wilde A.A.M., Faivre L., Ackerman M.J., Lodder E.M., Bezzina C.R. (2019). Supraventricular tachycardias, conduction disease, and cardiomyopathy in 3 families with the same rare variant in TNNI3K (p.Glu768Lys).. Heart Rhythm.

[30] Pham C., Martín M.N., Lodder E.M. (2021). The diverse roles of tnni3k in cardiac disease and potential for treatment.. Int. J. Mol. Sci..

[31] Calkins H., Sousa J., Atassi E.R., Rosenheck S., Buitleir d.M., Kou W.H., Kadish A.H., Langberg J.J., Morady F. (1991). Diagnosis and cure of the Wolff-Parkinson-White syndrome or paroxysmal supraventricular tachycardias during a single electrophysiologic test.. N. Engl. J. Med..

[32] Kay G.N., Epstein A., Dailey S.M., Plumb V.J. (1993). Role of radiofrequency ablation in the management of supraventricular arrhythmias: Experience in 760 consecutive patients.. J. Cardiovasc. Electrophysiol..

[33] Farshidi A., Josephson M.E., Horowitz L.N. (1978). Electrophysiologic characteristics of concealed bypass tracts: Clinical and electrocardiographic correlates.. Am. J. Cardiol..

[34] Cho J.G., Kim J.W., Ahn Y.K., Bae Y., Kim J.H., Kim S.H., Park J.H., Jeong M.H., Park J.C., Kang J.C. (1998). Radiofrequency catheter ablation in familial paroxysmal supraventricular tachycardia due to accessory atrioventricular pathways.. Jpn. Circ. J..

[35] Massumi R.A. (1967). Familial Wolff-Parkinson-White syndrome with cardiomyopathy.. Am. J. Med..

[36] Al-Khatib S.M., Pritchett E.L. (1999). Clinical features of Wolff-Parkinson-White syndrome.. Am Heart J.

[37] Chia B.L., Yew F.C., Chay S.O., Tan A.T.H. (1982). Familial wolff-parkinson-white syndrome.. J. Electrocardiol..

[38] Vidaillet H.J., Pressley J.C., Henke E., Harrell F.E., German L.D. (1987). Familial occurrence of accessory atrioventricular pathways (preexcitation syndrome).. N. Engl. J. Med..

[39] Sidhu J.S., Rajawat Y.S., Rami T.G., Gollob M.H., Wang Z., Yuan R., Marian A.J., DeMayo F.J., Weilbacher D., Taffet G.E., Davies J.K., Carling D., Khoury D.S., Roberts R. (2005). Transgenic mouse model of ventricular preexcitation and atrioventricular reentrant tachycardia induced by an AMP-activated protein kinase loss-of-function mutation responsible for Wolff-Parkinson-White syndrome.. Circulation.

[40] MacRae C.A., Ghaisas N., Kass S., Donnelly S., Basson C.T., Watkins H.C., Anan R., Thierfelder L.H., McGarry K., Rowland E. (1995). Familial hypertrophic cardiomyopathy with wolff-parkinson-white syndrome maps to a locus on chromosome 7q3.. J. Clin. Invest..

[41] Murphy R.T., Mogensen J., McGarry K., Bahl A., Evans A., Osman E., Syrris P., Gorman G., Farrell M., Holton J.L., Hanna M.G., Hughes S., Elliott P.M., MacRae C.A., McKenna W.J. (2005). Adenosine monophosphate-activated protein kinase disease mimicks hypertrophic cardiomyopathy and Wolff-Parkinson-White syndrome.. J. Am. Coll. Cardiol..

[42] Gollob M.H., Seger J.J., Gollob T.N., Tapscott T., Gonzales O., Bachinski L., Roberts R. (2001). Novel PRKAG2 mutation responsible for the genetic syndrome of ventricular preexcitation and conduction system disease with childhood onset and absence of cardiac hypertrophy.. Circulation.

[43] Koneru J.N., Wood M.A., Ellenbogen K.A. (2012). Rare forms of preexcitation: A case study and brief overview of familial forms of preexcitation.. Circ. Arrhythm. Electrophysiol..

[44] Arad M., Maron B.J., Gorham J.M., Johnson W.H., Saul J.P., Atayde P.A.R., Spirito P., Wright G.B., Kanter R.J., Seidman C.E., Seidman J.G. (2005). Glycogen storage diseases presenting as hypertrophic cardiomyopathy.. N. Engl. J. Med..

[45] Tan H.L., van der Wal A.C., Campian M.E., Kruyswijk H.H., ten Hove Jansen B., Doorn v.D.J., Oskam H.J., Becker A.E., Wilde A.A.M. (2008). Nodoventricular accessory pathways in PRKAG2-dependent familial preexcitation syndrome reveal a disorder in cardiac development.. Circ. Arrhythm. Electrophysiol..

[46] Cheung P.C., Salt I.P., Davies S.P., Hardie D.G., Carling D. (2000). Characterization of AMP-activated protein kinase gamma-subunit isoforms and their role in AMP binding.. Biochem. J..

[47] Kübler W., Schömig A., Senges J. (1985). The conduction and cardiac sympathetic systems: Metabolic aspects.. J. Am. Coll. Cardiol..

[48] Arad M., Moskowitz I.P., Patel V.V., Ahmad F., Atayde P.A.R., Sawyer D.B., Walter M., Li G.H., Burgon P.G., Maguire C.T., Stapleton D., Schmitt J.P., Guo X.X., Pizard A., Kupershmidt S., Roden D.M., Berul C.I., Seidman C.E., Seidman J.G. (2003). Transgenic mice overexpressing mutant prkag2 define the cause of wolff-parkinson-white syndrome in glycogen storage cardiomyopathy.. Circulation.

[49] Light P.E., Wallace C.H.R., Dyck J.R.B. (2003). Constitutively active adenosine monophosphate-activated protein kinase regulates voltage-gated sodium channels in ventricular myocytes.. Circulation.

[50] Lalani S.R., Thakuria J.V., Cox G.F., Wang X., Bi W., Bray M.S., Shaw C., Cheung S.W., Chinault A.C., Boggs B.A., Ou Z., Brundage E.K., Lupski J.R., Gentile J., Waisbren S., Pursley A., Ma L., Khajavi M., Zapata G., Friedman R., Kim J.J., Towbin J.A., Stankiewicz P., Schnittger S., Hansmann I., Ai T., Sood S., Wehrens X.H., Martin J.F., Belmont J.W., Potocki L. (2008). 20p12.3 microdeletion predisposes to Wolff-Parkinson-White syndrome with variable neurocognitive deficits.. J. Med. Genet..

[51] Weyhrauch D.L., Ye D., Boczek N.J., Tester D.J., Gavrilova R.H., Patterson M.C., Wieben E.D., Ackerman M.J. (2016). Whole exome sequencing and heterologous cellular electrophysiology studies elucidate a novel loss-of-function mutation in the cacna1a-encoded neuronal p/q-type calcium channel in a child with congenital hypotonia and developmental delay.. Pediatr. Neurol..

[52] Wising P. (1941). Familial, Congenital Sinus Tachycardia.. Acta Med. Scand..

[53] Baruscotti M., Bucchi A., Milanesi R., Paina M., Barbuti A., Ruscone G.T., Bianco E., Serdoz V.L., Cappato R., DiFrancesco D. (2017). A gain-of-function mutation in the cardiac pacemaker HCN4 channel increasing cAMP sensitivity is associated with familial Inappropriate Sinus Tachycardia.. Eur. Heart J..

[54] Baruscotti M., Bianco E., Bucchi A., DiFrancesco D. (2016). Current understanding of the pathophysiological mechanisms responsible for inappropriate sinus tachycardia: Role of the If "funny" current.. J. Inter. Card Electro..

[55] Still A.M., Huikuri H., Airaksinen K.J., Koistinen M.J., Kettunen R., Hartikainen J., Mitrani R.D., Castellanos A., Myerburg R.J., Raatikainen M.J.P. (2002). Impaired negative chronotropic response to adenosine in patients with inappropriate sinus tachycardia.. J. Cardiovasc. Electrophysiol..

[56] Nattel S. (2006). Inappropriate sinus tachycardia and beta-receptor autoantibodies: A mechanistic breakthrough?. Heart Rhythm.

[57] Ahmed A., Pothineni N.V.K., Charate R., Garg J., Elbey M., Asmundis d.C., LaMeir M., Romeya A., Shivamurthy P., Olshansky B., Russo A., Gopinathannair R., Lakkireddy D. (2022). Inappropriate sinus tachycardia: Etiology, pathophysiology, and management.. J. Am. Coll. Cardiol..

[58] Baruscotti M., Barbuti A., Bucchi A. (2010). The cardiac pacemaker current.. J. Mol. Cell. Cardiol..

[59] Chiale P.A., Garro H.A., Schmidberg J., Sánchez R.A., Acunzo R.S., Lago M., Levy G., Levin M. (2006). Inappropriate sinus tachycardia may be related to an immunologic disorder involving cardiac β andrenergic receptors.. Heart Rhythm.

[60] Cappato R., Castelvecchio S., Ricci C., Bianco E., Serdoz V.L., Ruscone G.T., Pittalis M., Ambroggi D.L., Baruscotti M., Gaeta M., Furlanello F., Francesco D.D., Lupo P.P. (2012). Clinical efficacy of ivabradine in patients with inappropriate sinus tachycardia: A prospective, randomized, placebo-controlled, double-blind, crossover evaluation.. J. Am. Coll. Cardiol..

[61] Scheinman M.M., Vedantham V. (2012). Ivabradine.. J. Am. Coll. Cardiol..

[62] Olshansky B., Sullivan R.M. (2013). Inappropriate sinus tachycardia.. J. Am. Coll. Cardiol..

[63] Pellegrini C.N., Scheinman M.M. (2016). Epidemiology and definition of inappropriate sinus tachycardia.. J. Interv. Card. Electrophysiol..

[64] Li Z., Wang Q., Sun X., Zhang Y., Cui C., Chen H., Chen M. (2023). Atrial tachycardia with concomitant prolonged hv interval with an scn5a missense variant (p.r367h).. JACC Clin. Electrophysiol..

[65] Hayakawa I., Abe Y., Ono H., Kubota M. (2019). Severe congenital RYR1-associated myopathy complicated with atrial tachycardia and sinus node dysfunction: A case report.. Ital. J. Pediatr..

[66] Theis J.L., Zimmermann M.T., Larsen B.T., Rybakova I.N., Long P.A., Evans J.M., Middha S., Andrade d.M., Moss R.L., Wieben E.D., Michels V.V., Olson T.M. (2014). TNNI3K mutation in familial syndrome of conduction system disease, atrial tachyarrhythmia and dilated cardiomyopathy.. Hum. Mol. Genet..

[67] Dagres N., Gutersohn A., Wieneke H., Sack S., Erbel R. (2004). A new hereditary form of ectopic atrial tachycardia with autosomal dominant inheritance.. Int. J. Cardiol..

[68] Balaji S., Sullivan I.D., Shinebourne E.A. (1996). Familial neonatal atrial tachycardia.. Heart.

[69] Brodsky M., Wu D., Denes P., Rosen K.M. (1977). Familial atrial tachyarrhythmia with short PR interval.. Arch. Intern. Med..

[70] Zhao Y., Meng X.M., Wei Y.J., Zhao X.W., Liu D.Q., Cao H.Q., Liew C.C., Ding J.F. (2003). Cloning and characterization of a novel cardiac-specific kinase that interacts specifically with cardiac troponin I.. J. Mol. Med..

[71] Milano A., Lodder E.M., Bezzina C.R. (2015). TNNI3K in cardiovascular disease and prospects for therapy.. J. Mol. Cell. Cardiol..

[72] Benson D.W., Wang D.W., Dyment M., Knilans T.K., Fish F.A., Strieper M.J., Rhodes T.H., George A.L. (2003). Congenital sick sinus syndrome caused by recessive mutations in the cardiac sodium channel gene (SCN5A).. J. Clin. Invest..

[73] Gellens M.E., George A.L., Chen L.Q., Chahine M., Horn R., Barchi R.L., Kallen R.G. (1992). Primary structure and functional expression of the human cardiac tetrodotoxin-insensitive voltage-dependent sodium channel.. Proc. Natl. Acad. Sci. USA.

[74] Asseman P., Berzin B., Desry D., Vilarem D., Durand P., Delmotte C., Sarkis E.H., Lekieffre J., Thery C. (1983). Persistent sinus nodal electrograms during abnormally prolonged postpacing atrial pauses in sick sinus syndrome in humans: Sinoatrial block *vs* overdrive suppression.. Circulation.

[75] Asseman P., Berzin B., Desry D., Bauchart J.J., Reade R., Leroy O., Poncelet P., Lekieffre J., Thery C. (1991). Postextrasystolic sinoatrial exit block in human sick sinus syndrome: Demonstration by direct recording of sinus node electrograms.. Am. Heart J..

[76] Villain E., Vetter V.L., Garcia J.M., Herre J., Cifarelli A., Garson A. (1990). Evolving concepts in the management of congenital junctional ectopic tachycardia. A multicenter study.. Circulation.

[77] Alasti M., Mirzaee S., Machado C., Healy S., Bittinger L., Adam D., Kotschet E., Krafchek J., Alison J. (2020). Junctional ectopic tachycardia (JET).. J. Arrhythm..

[78] Dubin A.M., Cuneo B.F., Strasburger J.F., Wakai R.T., Hare v.G.F., Rosenthal D.N. (2005). Congenital junctional ectopic tachycardia and congenital complete atrioventricular block: A shared etiology?. Heart Rhythm.

[79] Xi Y., Honeywell C., Zhang D., Schwartzentruber J., Beaulieu C.L., Tetreault M., Hartley T., Marton J., Vidal S.M., Majewski J., Aravind L., Gollob M., Boycott K.M., Gow R.M. (2015). Whole exome sequencing identifies the TNNI3K gene as a cause of familial conduction system disease and congenital junctional ectopic tachycardia.. Int. J. Cardiol..

[80] Wheeler F.C., Tang H., Marks O.A., Hadnott T.N., Chu P.L., Mao L., Rockman H.A., Marchuk D.A. (2009). Tnni3k modifies disease progression in murine models of cardiomyopathy.. PLoS Genet..

[81] Azam M., Seeliger M.A., Gray N.S., Kuriyan J., Daley G.Q. (2008). Activation of tyrosine kinases by mutation of the gatekeeper threonine.. Nat. Struct. Mol. Biol..

[82] Eyers P.A., Craxton M., Morricel N., Cohen P., Goedert M. (1998). Conversion of SB 203580-insensitive MAP kinase family members to drug-sensitive forms by a single amino-acid substitution.. Chem. Biol..

[83] Joseph R.E., Andreotti A.H. (2011). Controlling the activity of the Tec kinase Itk by mutation of the phenylalanine gatekeeper residue.. Biochemistry.

[84] Takeshima H., Komazaki S., Nishi M., Iino M., Kangawa K. (2000). Junctophilins: A novel family of junctional membrane complex proteins.. Mol. Cell.

[85] Landstrom A.P., Beavers D.L., Wehrens X.H.T. (2014). The junctophilin family of proteins: From bench to bedside.. Trends Mol. Med..

[86] Oort v.R.J., Garbino A., Wang W., Dixit S.S., Landstrom A.P., Gaur N., Almeida D.A.C., Skapura D.G., Rudy Y., Burns A.R., Ackerman M.J., Wehrens X.H.T. (2011). Disrupted junctional membrane complexes and hyperactive ryanodine receptors after acute junctophilin knockdown in mice.. Circulation.

[87] Yang Q., Tadros H.J., Sun B., Bidzimou M.T., Ezekian J.E., Li F., Ludwig A., Wehrens X.H.T., Landstrom A.P. (2023). Junctional ectopic tachycardia caused by junctophilin-2 expression silencing is selectively sensitive to ryanodine receptor blockade.. JACC Basic Transl. Sci..

[88] Landstrom A.P., Yang Q. (2023). Reduction in junctophilin 2 expression in cardiac nodal tissue results in intracellular calcium-driven increase in nodal cell automaticity.m. Circ. Arrhythm. Electrophysiol..

[89] Catterall W.A. (2000). From ionic currents to molecular mechanisms: The structure and function of voltage-gated sodium channels.. Neuron.

[90] Balser J. (1999). Structure and function of the cardiac sodium channels.. Cardiovasc. Res..

[91] Roberts R., Brugada R. (2003). Genetics and arrhythmias.. Annu. Rev. Med..

[92] Koitabashi N., Kass D.A. (2012). Reverse remodeling in heart failure—mechanisms and therapeutic opportunities.. Nat. Rev. Cardiol..

[93] Dorn G.W., Force T. (2005). Protein kinase cascades in the regulation of cardiac hypertrophy.. J. Clin. Invest..

[94] Darbar D., Roden D.M. (2006). Pharmacogenetics of antiarrhythmic therapy.. Expert Opin. Pharmacother..

